# Supplementary material for: Correlation Between Instrumented Timed Up and Go Test Performance and Muscle Strength in Knee and Hip Osteoarthritis: An Exploratory, Cross-Sectional Study
Source: Sensors (Basel). 2025 Dec 25;26(1):142. doi: 10.3390/s26010142 (PMC12787561; doi:10.3390/s26010142)
Supplement: Supplementary file 1 [file sensors-26-00142-s001.zip › sensors-3974310-supplementary.pdf]

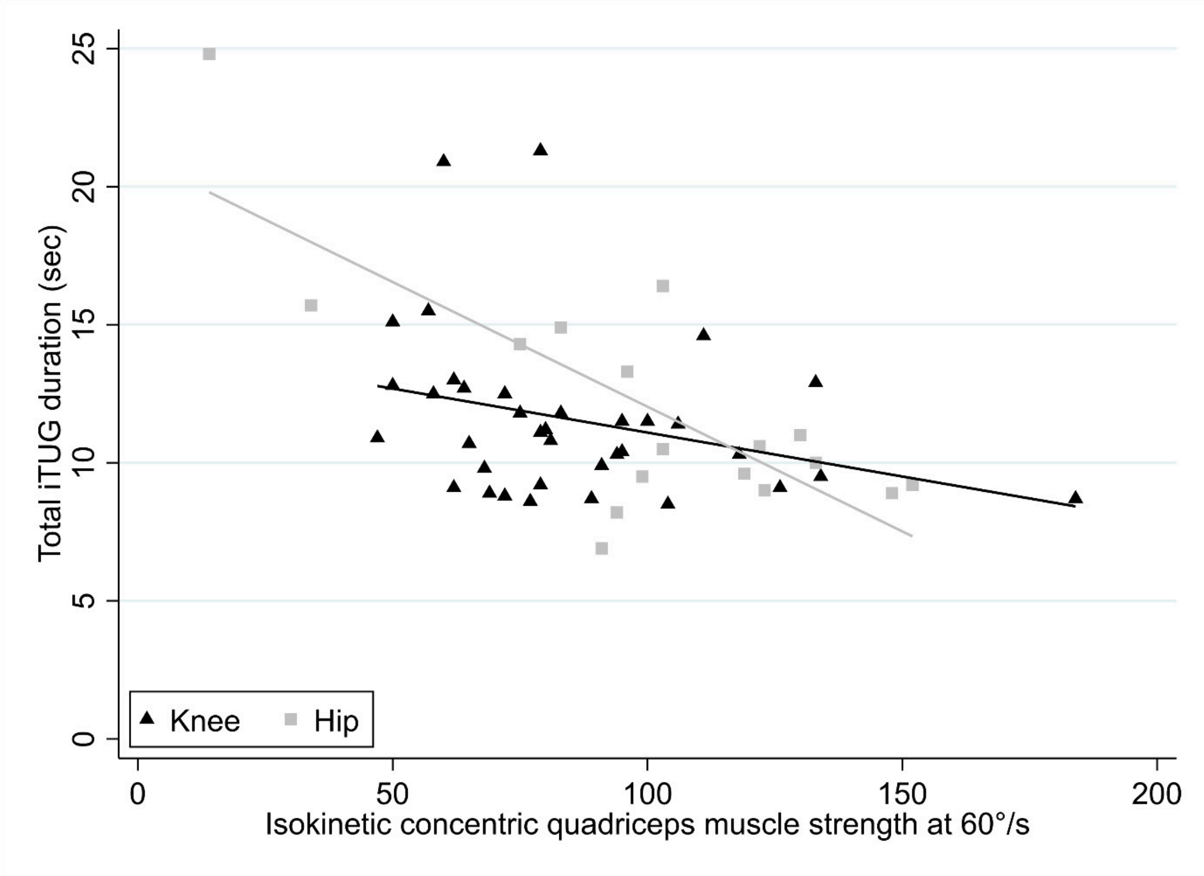

Supplementary Figure S1. Scatter plot illustrating the Spearman correlation between isokinetic concentric quadriceps muscle strength at 60°/s (normalized to body mass) and total iTUG duration in patients with hip and knee osteoarthritis.
